# Critical review of polymer and hydrogel deposition methods for optical and electrochemical bioanalytical sensors correlated to the sensor’s applicability in real samples

**DOI:** 10.1007/s00216-022-04363-2

**Published:** 2022-10-24

**Authors:** Meike Bauer, Axel Duerkop, Antje J. Baeumner

**Affiliations:** 1 Institute of Analytical Chemistry, Chemo- and Biosensors, University of Regensburg, 93040 Regensburg, Germany; 2 Department of Biological and Environmental Engineering, Cornell University, Ithaca, NY 14853 USA

**Keywords:** Optical and electrochemical (bio)sensors, Hydrogel, Polymer membrane, Deposition techniques

## Abstract

Sensors, ranging from in vivo through to single-use systems, employ protective membranes or hydrogels to enhance sample collection or serve as filters, to immobilize or entrap probes or receptors, or to stabilize and enhance a sensor’s lifetime. Furthermore, many applications demand specific requirements such as biocompatibility and non-fouling properties for in vivo applications, or fast and inexpensive mass production capabilities for single-use sensors. We critically evaluated how membrane materials and their deposition methods impact optical and electrochemical systems with special focus on analytical figures of merit and potential toward large-scale production. With some chosen examples, we highlight the fact that often a sensor’s performance relies heavily on the deposition method, even though other methods or materials could in fact improve the sensor. Over the course of the last 5 years, most sensing applications within healthcare diagnostics included glucose, lactate, uric acid, O_2_, H^+^ ions, and many specific metabolites and markers. In the case of food safety and environmental monitoring, the choice of analytes was much more comprehensive regarding a variety of natural and synthetic toxicants like bacteria, pesticides, or pollutants and other relevant substances. We conclude that more attention must be paid toward deposition techniques as these may in the end become a major hurdle in a sensor’s likelihood of moving from an academic lab into a real-world product.

**Graphical abstract:**

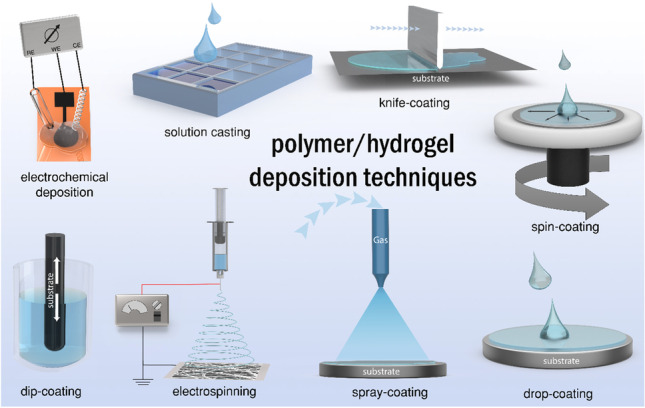

## Overview of membrane deposition techniques

Polymers and hydrogels play an essential role as a generally termed membrane in a majority of (bio)sensors as they facilitate recognition or receptor element immobilization, protection against negative matrix effects, pre-concentration of the analyte molecules, and prevention of interfering signals. Polymers and hydrogels can be classified by their properties like the network’s charge or by categories like their origin. Some of the hydrogels are derived from natural sources like polysaccharides (e.g., alginates or chitosan derivatives), or protein-based polymers like collagen [1]. Prominent examples for hydrogels assembled from synthetic building blocks are polyethylene glycol (PEG), polyvinyl chloride (PVC), and polyurethanes (PU) like Hydromed® D4 or Nafion®, a perfluorinated polymer which is often used as cation-selective conductive membrane [2, 3]. Depending on the aspired application, biocompatibility, mechanical or chemical stability, and fouling or degradation properties of the used polymers and hydrogels must be considered.

While the importance of the material is obvious, it is less known that the deposition method itself has an as relevant effect on the performance of the sensor. It influences the surface morphology, density and thickness of the film, and its attachment to the substrate. At the same time, the deposition technique is affected by the substrate, the solvents used, receptor elements, and the polymers and their concentration within the precursor cocktail. Most important techniques include drop coating and solution casting; knife, blade, or bar coating; spray coating; dip coating; spin coating; electrospinning; and electrochemical deposition. Another challenging method used in sensor and biosensor development which has to be mentioned but is not explicitly reviewed within the document is plasma polymerization [4, 5] especially in combination with molecular imprinting [6, 7]. It is not further addressed in this manuscript because of its highly specialized nature and limited use. Each reviewed method comes with unique parameters influencing the performance of the membrane (Table 1), so that it is advisable to carry out a thorough evaluation of the materials and receptor elements used with respect to the immobilization techniques and fabrication methods to optimize the resulting sensor. Combinations of different techniques and materials are a common strategy to overcome disadvantages and exploit the beneficial effects of the individual polymer and deposition method. Considering the purpose of sensors as mass product, their original academic development ought to keep a later mass production in mind. This also holds true for membrane deposition methods as some are significantly better suited for upscaling compared to others.

**Table 1:** Overview of herein reviewed polymeric film deposition techniques with their most important key facts as well as advantages and disadvantages to enable a suitable choice of a fitting technique for sensor development

Technique	Principle	Simplicity and apparatus effort	Film morphology	Potential for commercialization	Advantages	Disadvantages	Ref
Drop coating	Defined volume of polymeric cocktail is transferred with a pipette onto the substrate and dried/annealed	Very simple, very small instrumental demand, no special equipment needed, pipette is sufficient	No special morphology but self-assembling layers can be fabricated, no multilayered membranes	Roll-to-roll fabrication possible with large plotters, but also batch production	☑ Easiest method for proof of sensor concept ☑ For small and defined areas ☑ Simple and fast ☑ No waste	☒ Coffee-ring effect ☒ Reproducibility regarding film morphology and thickness	[8–12]
Solution casting	The polymeric cocktail is poured in a mold or special form and then dried/annealed	Medium simplicity, small apparatus effort, molds and forms are needed	Special morphology can be obtained by (nano)structured molds, thickness adjustable	Batch production for defined shapes, but also continuous process possible	☑ Special forms and shapes accessible without substrate ☑ Almost no waste	☒ Laborious and time consuming since more steps are necessary for multilayered sensors ☒ Preparing of the mold is required previously	[13, 14]
Knife coating	The polymeric cocktail is spread with a knife, blade, bar, or rod with defined distance over the substrate and dried/annealed	Simple, small apparatus effort, can be realized with very simple tools like tape or wire and doctor’s blade/bar/rod	No special morphology, smooth and even surface, easy access of multilayered membranes, thickness adjustable	Roll-to-roll fabrication already standard method	☑ Large areas can be coated fast and easy ☑ Low waste	☒ Reproducibility on lab scale ☒ Membrane thickness is restricted to spacer material or must be adjusted by sensor cocktail composition ☒ Sub-micron layers difficult to achieve	[15–18]
Spray coating	The polymeric cocktail is applied as very small droplets via pressurized air/gases through a nozzle on the substrate	Simple, medium apparatus effort, spray chamber with aspiration, pressured air/gas supply for even and uniform spray mist	No special morphology, smooth and even surface, self-assembling layers and layer-by-layer buildups can be produced easily, thickness adjustable from nm to µm	Roll-to-roll fabrication already standard method but also batch production possible	☑ Easy layer-by-layer deposition ☑ For large areas ☑ Special forms and shapes easily accessible ☑ Contactless	☒ For small areas, masks or templates required ☒ Waste of precious sensor cocktail ☒ Harmful aerosols may be formed ☒ Nozzle clogging and cleaning	[19–22]
Dip coating	The substrate is dipped into the polymeric cocktail with subsequent annealing/drying	Very simple, no apparatus effort, simply a suitable vessel	No special morphology, smooth and even surface, thickness adjustable from nm to µm, easy access of multilayered membranes	Roll-to-roll fabrication already standard method but also batch production possible	☑ Very simple and easy ☑ Sophisticated methods available for precise film control	☒ Waste of material by coating of unwanted areas like backside or masks with simple methods	[23–25]
Spin coating	An excess polymeric cocktail is placed in the middle of the substrate which is rotated fast at a defined speed to spread the polymeric solution homogeneously by centrifugal forces	Simple, medium apparatus effort, a commercial or in-house-made spin-coating device with adjustable reproducible spinning speed is necessary	No special morphology, smooth and even surface, thickness adjustable, better for thin membranes, multilayering difficult to achieve	Just batch production and no continuous approach	☑ Smooth and even films on lab scale and industrial scale	☒ Waste of material since an excess must be applied on the substrate ☒ Reproducibility	[26–28]
Electro spinning	The polymeric cocktail is deposited in the form of nanofibers via electrical potential difference between a spray nozzle and a grounded collector substrate	Medium simplicity, large apparatus effort, high-voltage supply safety restrictions, pump with very homogeneous feed rate	Very defined surface morphology, porous networks and structures can be realized easily, thickness adjustable from nm to µm, easy access of multilayered membranes	Batch-to-batch production and continuous process, but still too slow for market-relevant applications on industrial scale	☑ Conductive and non-conductive substrates possible ☑ Very high surface-to-volume ratio of deposited film	☒ Apparatus effort and safety requirements ☒ Worse reproducibility on lab scale ☒ Equipment more expensive	[29–31]
Electrochemical deposition	The polymeric precursor cocktail is applied on the conductive substrate, and a voltage/current is applied to start the polymerization process by reduction/oxidation of the precursors	Simple, medium apparatus effort, potentiostat and RE (e.g., Ag/AgCl) and/or CE (e.g., Pt wire) is necessary	Surface morphology can be adjusted by electrical settings, thickness adjustable, very good tor thin layers, easy access to multilayered membranes	Batch-to-batch production thinkable	☑ Deposition occurs just on defined area even if cocktail is not applied exactly ☑ Highly reproducible also on lab scale	☒ Just suitable for conductive and semiconductive substrates	[11, 12, 32, 33]

### Drop coating

Drop coating or drop casting is the most simple and fast technique to deposit a polymeric layer on a surface and modify it with a receptor element. Especially on lab scale, it is the easiest approach for surface modification that generates essentially no waste material. On the industrial scale, this technique can be realized by large plotters. It is best suited for coating of a small and defined area, because for larger areas, controlling thickness, porosity, and uniformity of the film is more difficult [34]. The general process includes the mixing of recognition elements such as enzymes [8, 9, 35, 36], DNA derivatives [10, 37], or probes such as fluorescent dyes, luminophores [38], or nanoparticles [39, 40] with an evaporable solvent and a binder (e.g., hydrogels, polymers, or cross-linkers like glutaraldehyde), followed by application of this cocktail to the desired surface. In addition to the cocktail composition and surface conditions, drying time, annealing temperature, and the applied volume are contributing factors toward the final homogeneity and morphology of the deposited material. Here, the coffee-ring-effect phenomenon presents a significant limitation on the reproducibility of drop-casted surfaces and requires partly complex strategies to be overcome [41]. While mainly organic solvents and binders are used, water-based solvents are needed for the entrapment of fragile biological molecules such as enzymes.

**Fig. 1:**
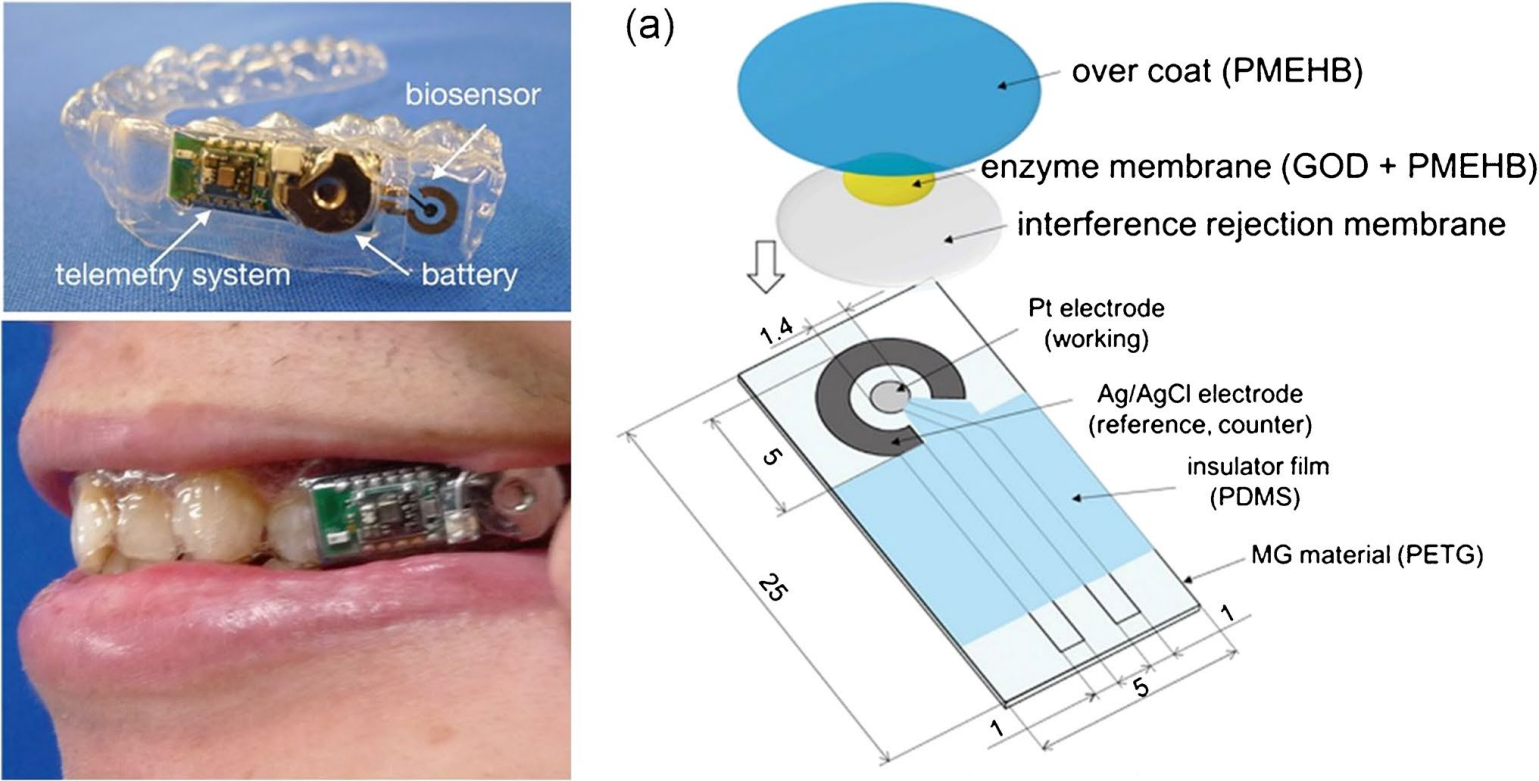
Arakawa et al. demonstrated a biocompatible glucose sensor placed within a mouthguard using the straightforward drop-coating method since film thickness and morphology are irrelevant for the performance (reprinted with permission from [44]; Copyright © 2020 American Chemical Society)

The sheer simplicity of the approach ensures widespread use with mixtures based on Nafion, chitosan (CS), cellulose acetate (CA), or conducting polymers like poly(3,4-ethylenedioxythiophene) (PEDOT) and other hydrogels and polymers for electrochemical detection of glucose [8, 9], lactate [35], and uric acid [42] in different body fluids like sweat, blood, and tears; tetrodotoxin in seafood samples [39]; heavy metals in wastewater [9]; biogenic amines in food samples [43]; HIV-1-gene in blood [10]; and pH values of various aqueous solutions [38]. In these examples, relevant improvements to the drop-casting method include doping of Nafion with graphene to improve dispersion and subsequent electrochemical sensing [10], and layer-by-layer assemblies of, e.g., chitosan/Nafion/ionic liquid/ferrocene composite film on top of a carbon electrode [8]. It should be pointed out that the right selection of polymer in relationship to the analyte of interest is of utmost importance. The described detection of heavy metals using chitosan as polymer layer should see significant improvement, if neutral polymers are chosen instead [11]. Arakawa et al. demonstrate a biocompatible sensor placed within mouthguards (Fig. 1) where drop casting is the method of choice since the film thickness is irrelevant for the sensor performance [44].

### Solution casting

For solution casting methods, the polymeric solution can be poured and dried in a mold or between glass plates to obtain a defined thickness or shape. Film morphology and its quality in general mainly depend on the homogeneity of the cocktail, its concentration, solvents used, temperature, and pressure applied during evaporation or annealing [34, 45]. It is a common technique for casting PDMS generating specific sensor shapes with low demands toward the substrate. Importantly, casting of larger areas can be accomplished on an industrial scale, but just as a batch process. Continuous approaches are difficult for film productions but have been demonstrated successfully for castings of small molds in special shapes.

Single-layered approaches are very straightforward, e.g., Gasper et al. exploits the high thermal conductivity and stability of molded PDMS for an Eu(III) β-diketonate complex–based temperature sensor. Since the luminescent optical probe is highly soluble within the polymeric cocktail, inhomogeneities within the casted sensor are easily avoided [13]. Multilayered approaches require more intricate casting strategies. Bartelmess et al. [14] developed a bi-layered sensing optode with ratiometric fluorescence readout for the monitoring of corrosion in concrete. The preparation process is laborious and time consuming due to the different drying, and multiple mold-filling steps. Yet, it is currently the only technique to obtain such specialized sensor shapes and further ingenuity is needed to bring it from a lab-scale proof of concept amenable toward a mass production practice.

### Knife coating

Knife coating, also known as spread coating, bar coating, or blade coating, is a simple and fast coating technique for large areas on a flexible substrate without a defined surface pattern [34]. This technique can easily be adapted to industrial scale (role-to-role fabrication) which enables a continuous and cost-effective high-throughput production [46, 47]. Usually, the knife or blade is fixed, and the substrate is moved underneath at a certain distance to define the wet layer thickness. There is low waste of the coating material, and therefore, the method is also useful for expensive coatings. On lab scale, typically the supporting material is fixed, and the blade or knife is moved with a defined gap over the substrate [47]. Other approaches use a bar [15] or rod twined around with a wire of stated thickness to define the gap between the substrate and the moving part, and subsequently the layer thickness [45]. Tape or other spacer materials are similarly used at lab scale to define the distance between substrate and blade. Commonly, the resulting film thickness can be regulated by the gap size between substrate and knife, the viscosity of the coated cocktail which is mainly influenced by its composition and temperature, the coating speed, surface tension and wetting properties of the substrate and the amount of volatile solvent contained in the cocktail [46, 47]. Therefore, cleaning and perhaps a pre-treatment step of the substrate is necessary to obtain a smooth, even, and lasting film. But most critically, this method is unsuitable for making sub-microscale films, and furthermore, controlling the micrometric precision of the blade is difficult or restricted by the thickness of the used spacers [34, 48]. Furthermore, drying or annealing processes after the actual deposition process can influence the film building and must be optimized or automated to obtain reproducible films.

However, knife coating has received the least attention in electrode fabrication but is a low-cost and straightforward process for fabrication of optical sensor membranes like many research groups showed. Important examples are optical sensors for pH [15, 16], oxygen [16, 49], gaseous sulfur mustard [17], and ammonia [18]. Many groups use Hydromed D4, a polyurethane-based hydrogel as 5 wt% or 10 wt% solution in ethanol/water mixtures for knife coating on flexible substrates due to its superb film-building properties [15, 17, 18], but also polystyrene [49] and Nafion [15] solutions are suitable candidates for knife coating. The active components are dissolved or suspended homogeneously within the polymeric solution. This cocktail is then coated with a defined layer thickness to obtain even and homogeneous films without special surface morphology containing enzymes, probes, or fluorescent dyes. Additional cross-linking agents like glutaraldehyde (GA) fix soluble components within the hydrogel network by covalent cross-linking. Additionally, GA cross-linking can also serve to form molecular imprints in the polymer to form unique biomimetic materials working as receptors for recognition and binding of target molecules [50, 51].

Maierhofer et al. investigated knife-coated dual-lifetime referencing ammonia sensors with tunable sensitivity and limit of detection (LOD) based on the respective hydrogel/polymer mixtures [18]. Polymers, solvents, and dyes are perfectly balanced regarding hydrophilicity that allow coating layer by layer without influencing the lower layer. The hydrophobic layer on top of the membrane ensemble enables the gaseous compounds to enter but prevents the recognition elements from leaching. The reference dye in each sensor membrane overcame the not optimum reproducibility of layer thickness for each coated sensor foil. Jiang et al. harnessed knife coating to obtain high spatial resolution and improved a similar oxygen sensor system by introduction of an optical isolation layer containing carbon black to minimize wavelength-dependent backscattering and reflections from any background [16]. This strategy can be found in many optical sensors applied to real-world samples.

Dalfen et al. demonstrated bar coating for composite films. D4 was chosen to provide a near-aqueous environment for the entrapped pH-sensitive diazaoxotriangulenium (DAOTA) dyes whereas Nafion virtually eliminates the negative influence of anions like chloride and nitrate [15]. Since the highly charged matrix affected the p*K*_a_ values of the embedded dye negatively, the group concluded that covalent attachment to the polymer support may be needed. This suggests though that another membrane deposition method must be chosen to enable high surface-to-volume ratios to provide high dye-immobilization densities. Also, Tribuser et al. demonstrated how properties like sensitivity or quantum yield of a K^+^ fluoroionophore change depending on the chosen polyurethane-based hydrogel matrix with different hydrophilicity using the knife-coating technique for film preparation [52].

Bidmanova et al. demonstrated that knife coating is highly suitable for the deposition of polymers onto sensing materials [17]. Specifically, commercially available pH stripes were layered with D4 fixed with GA vapor to prevent probe leakage and to enhance the long-term stability. The haloalkane dehalogenase LinB was co-immobilized with bovine serum albumin (BSA) using different techniques. While this proof of principle could have been accomplished using drop coating on lab scale, the demonstrated knife coating suggests the applicability for mass production especially due to the commercial pH stripes and the polymer material chosen.

### Spray coating

Spray coating is a commonly used, simple and low-cost technique for the deposition of films in large areas. It can be performed in batch production on lab and industrial scales or as a roll-to-roll process in industry. It is a contactless deposition procedure that makes it an optimal coating process for sensitive substrate surfaces and materials. The coating fluid is atomized to droplets within a spray nozzle by pressurized air or gases like nitrogen or argon, and transferred on the substrate [45–47].

Although it is a simple method, many process parameters are crucial to determine surface morphology and layer thicknesses like nozzle configurations, pressure and composition of the carrier gas, coating speed, work distance, temperature, and number of sprayed layers [19–21, 34, 46]. Furthermore, the liquid properties of the coating solution or suspension like surface tension, viscosity, density, and vapor pressure influence the quality of the sprayed coating layer [34, 53]. Disadvantages could be harmful exposure to the aerosols of the spray mist and the difficulty of preventing the nozzle from clogging which requires a sophisticated and careful cleaning process of the nozzles. The method is especially useful for the coating of full and large areas. When masks or templates are used, much waste may be produced and, often, low edge resolution is observed [46, 53]. On the other hand, complicated sensor shapes become accessible, easily. As a main advantage, the method enables a simple generation of thick films via layer-by-layer applications [20, 54].

**Fig. 2:**
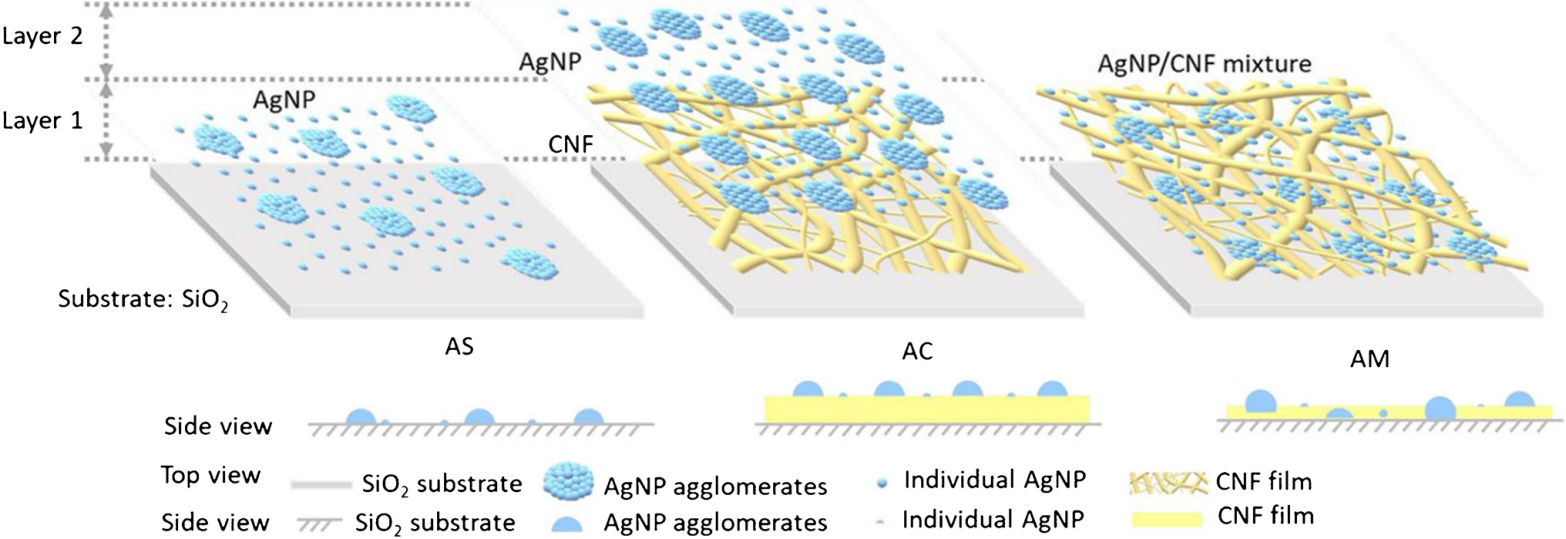
Chen et al. presented the self-assembling of spray-coated Ag nanoparticles (AgNP) on blank SiO_2_ substrate (AS), with cellulose nanofibrils (CNFs) as bottom layer (AC), and applying both within a mixture (AM) on the SiO_2_ substrate. They demonstrate the modification of the surface contact angle and suppose their potential as antifouling coating or use as label-free biosensors. (Reprinted with permission from [21], Copyright © 2021 The Authors. Published under a Creative Common Attribution CC-BY License)

Still, especially as proof-of-principle applications, interesting concepts for advanced spray coating have been published recently. This includes a fully flexible electrode array using MXene-polypyrrole nanowire mixtures as interconnecting components [22], and a thick and uniform poly(3,4-ethylenedioxythiophene) polystyrene sulfonate (PEDOT:PSS) layer as organic electrochemical transistor for monitoring electrophysiological activities [20], which turned out to be more successful than using spin coating. Chen et al. investigated spray coating for silver nanoparticle composites [21], where especially a layer-by-layer approach supported superior morphology and self-assembly of AgNPs mixed with cellulose (Fig. 2). Considering that AgNP coating is also used for hydrophilic antifouling coverings and label-free biosensors, this finding should have far-reaching effects. Finally, even thin graphene oxide (GO) films could be spray coated where the low substrate heating temperature preserves most of the oxygen-containing functional groups suggesting it to be an optimal method for GO film generation [19].

### Dip coating

Dip coating is a common technique used on lab and industrial scale for thin-film coatings and involves four stages: immersion, dwelling, withdrawal, and drying [45]. The surface morphology and thickness of the layer are influenced mainly by the properties of the dip solution and the substrate to be treated, like similar polarity, and furthermore from process parameters like process temperature, dwelling time, dipping and withdrawal speed, and finally drying time and temperature. The method does not require any special equipment [45] and can also be carried out as a batch or continuous process on the industrial scale [55]. Preferably, controlled conditions are applied since the method is susceptible to defects caused by contamination, aggregation of precursors, microscopic air bubbles in the solution, and irregularities in the supporting substrate surface [56]. Repeating the process several times can also minimize the defects but results in increased thickness. Dip coating can coat membranes and substrates by adding layers with 100 nm to 100 μm thickness and with pore sizes ranging between 1 nm and 5 µm [56]. The best thin-film building can be observed for high-viscosity solutions and cocktails with high surface tension [46, 47].

While classical dip coating may waste material covering front and backsides of substrates, Ceratti et al. demonstrated a novel process coating large areas on just one side with a high uniformity [57], which also largely impacts multilayer processes. Further refined deposition of material is also possible as demonstrated by Xiong et al. [23] where the distal end of a fiber optic is appended with a carbon quantum dots/cellulose acetate (CQDs/CA) mixture enabling the formation of a highly adrenaline-sensitive sensor for continuous and real-time detection via fluorescence quenching within physiological relevant concentration ranges. Similarly, the end of a quartz fiber was dip coated with a CQDs/glucose oxidase (GOx)/CAcomposite material to obtain a highly selective glucose sensor [24]. Finally, also electrochemical concepts have been demonstrated such as the wearable motion sensors using a spandex strand dip coated with graphene nanoplatelets and shielded by silicon rubber that are used as electrical conductive yarn [25]. Common to all these approaches is the use of relatively inexpensive materials, the avoidance of material waste, and limiting the applications solely for single-layer coatings.

### Spin coating

Spin coating is a technique used for spreading a uniform thin-film layer on a substrate by centrifugal forces. The method can be performed on laboratory scale in small benchtop devices which is a fast and cheap method. On the industrial scale, spin coating is used in batch processes since it is not suitable for continuous roll-to-roll processes. In general, an excess amount of the solution is placed in the middle of the substrate and is rapidly spread during the spinning process to the edges of the fast-rotating substrate. Film thickness can be empirically controlled by spin speed, time, temperature, volume of added substrate, composition, and viscosity of the applied solution as well as the wetting properties of the substrate [34]. Spin coating can coat membranes with thickness in the range of 70 to 500 nm, and pore size varies continuously from 4 to 200 nm [56]. Reproducibility issues limit this technique to a few substrates and go along with some waste of the coating solution unless spun-off solution can be safely re-used [53]. Drying or annealing of the spin-coated material is necessary and influences the quality and thickness of the applied layer. A very flat substrate surface is required to obtain a homogeneous film thickness over the entire area and to avoid streaks. Usually, the spinning itself is done within seconds but the annealing and drying may take hours or days.

**Fig. 3:**
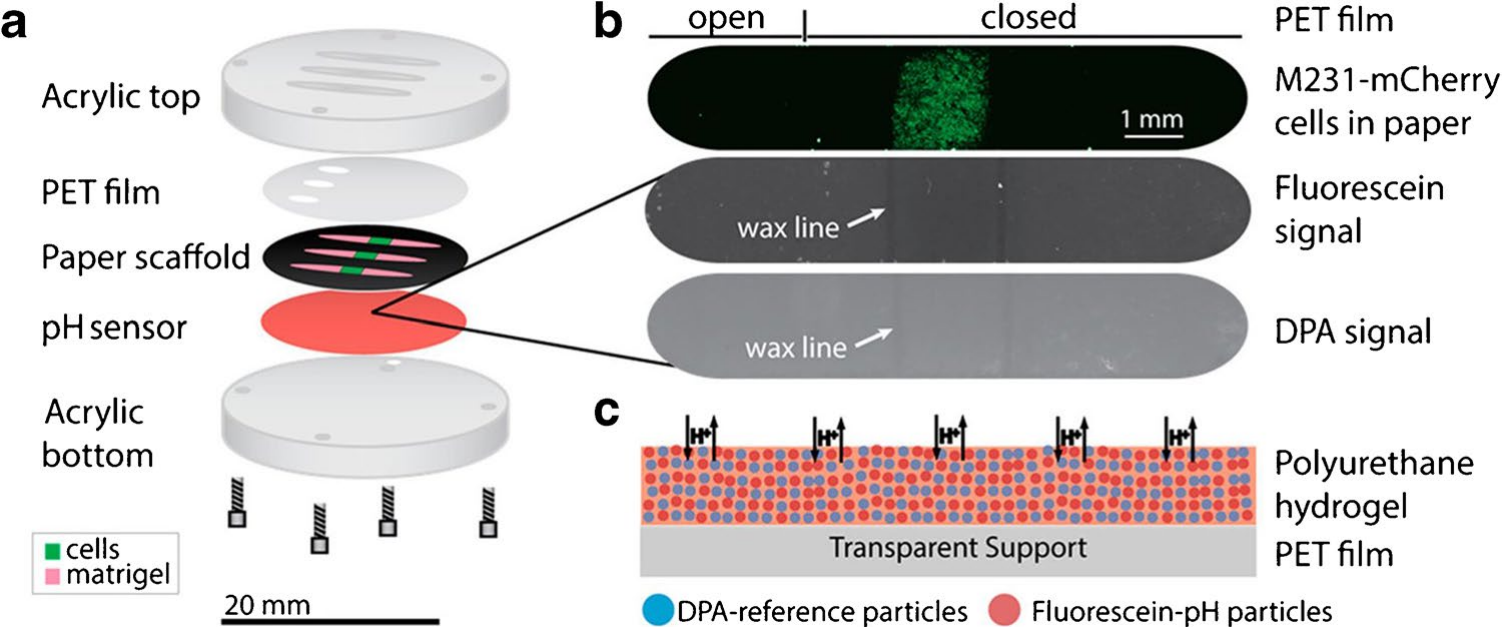
Kenney et al. presented an optical pH sensor for mapping spatiotemporal gradients in three-dimensional paper-based cell cultures. The D4 membrane contains the pH-sensitive fluorescein dye and diphenylanthracene (DPA) as reference dye. The respective polymer cocktail was spin coated on a transparent PET support (reprinted with permission from [27], Copyright © 2018 American Chemical Society)

Biring et al. demonstrated that specific and different spin coating can be accomplished on the two sides of a sensor substrate to result in an optical dual gas sensor for the simultaneous detection of oxygen and ammonia [26]. The oxygen-sensitive platinum porphyrin derivative complex was spin coated in ethyl cellulose on one side of a glass slide whereas on the backside the ammonia-sensitive eosin Y dye in cellulose acetate was applied. The D4 polymer was shown to also work well for spin coating by Kenney et al. developing an optical pH sensor for paper-based cell cultures (Fig. 3) [27]. Also, for electrochemical sensors, spin coating is advantageous including polymers and composite materials of polymers and nanoparticles. For example, Yoon et al. presented a flexible Kapton® polymer electrode with sputtered gold and spin-coated MoS_2_ nanoparticles with chemically bound GOx for glucose sensing [58]. The surface-sensitive surface-plasmon resonance (SPR) method has also relied on spin-coating processes where, for example, Gao et al. presented a sensor for uric acid detection. The group entrapped uricase in SiO_2_ mesoporous foams and SiNPs in a polyethylene glycol/polyvinyl alcohol (PEG/PVA) composite gel on a gold surface [28]. They emphasize that spin coating is the method of choice as a very flat and thin surface film can be created. This statement can be supported as dip-coated SPR sensors for uricase by Kant et al. [59] have a nearly two orders of magnitude higher LOD.

### Electrospinning

Electrospinning is an electrodynamic one-step process which uses electrical potential differences to produce ultrafine, long and continuous nanofibers with diameters at micro- to nanoscale on a conductive collector substrate [34, 45, 60]. Electrospun nanofibers are favorable for applications where a large and porous surface area with high functionalization ability is beneficial [61]. Therefore, electrospinning is a predestinated technique for sensor film coatings with subsequent immobilization steps. Beside other techniques for generating nanofiber networks on surfaces [60], electrospinning appears as a simple inexpensive process which is controllable via many process parameters like temperature, air humidity, potential, distance between collector and nozzle, and the properties of the spinning solution itself [62]. But on lab scale, it can be difficult to keep temperature and air humidity constant which are critical parameters for reproducibility. Furthermore, clogging of the polymeric solution within the syringe and nozzle must be avoided by optimizing the cocktail composition and parameters like feed speed. Electrospinning on a conductive but non-transparent material can lead to the necessity of an additional transfer step to a transparent substrate in case of optical approaches. Enzymes, dyes, nanoparticles, or other transducers can be directly entrapped within the fiber by dispersing or dissolving them within the spinning solution or can be afterwards immobilized either on the fiber surface or on top of the porous network by different techniques [61, 63]. Hardware requirements are more complex than for the other techniques regarding especially the high-voltage power supply safety restrictions [64]; however, a large number of natural and synthetic polymers can be spun, the morphology of the nanofibers and the collected mats can be tailored toward special features, and the method is easily scalable [65] albeit with still relatively slow production rates [66]. Through the spinning parameters, the thickness of the nanofibers can be influenced, but generating nanofibers with diameters below 10 nm is challenging [34]. Compared to the other methods, electrospinning enables the easy production of very thin films with a large surface-to-volume ratio due to the extended porosity [45]. Especially for gas-sensing devices, the high porosity leads to an unexpected increase in sensitivity compared to other materials [67]. Several analytical applications are described where doping of the polymer solution enables the immediate generation of as-use sensing nanofibers such as those made of cellulose acetate fibers doped with fluorescence probes where the high surface area afforded through the fiber structure lowered the LOD for biogenic amines by an order of magnitude in comparison to deposited films [29]. Similar findings were made for electrochemical ochratoxin detection [30]. Biocompatibility seems to be dependent on the polymer and solvents used as for other film deposition methods, and post-modification for immobilization of recognition elements can be performed likewise [31].

### Electrochemical deposition

Electrochemical deposition, also known as electrodeposition, electrophoretic deposition, or electroplating, is a traditional and inexpensive process used for thin-film coating of polymers and metal-based nanostructures on a conductive or semiconductive support such as indium tin oxide, gold, or carbon-based materials by an electrical current or redox reaction [34, 56]. It has been the method of choice for the functionalization of electrochemical sensors and deposition of conductive polymers such as poly(acetylene), PEDOT, poly(thiophene), poly(p-phenylene vinylene), poly(pyrrole), and poly(aniline) [68–70]. A simple two- or three-electrode setup is dipped into the precursor solution. An appropriate potential is applied, typically through cyclic voltammetry or chronoamperometry causing sufficient current flow to initiate the polymerization of the polymeric precursors directly on the electrode surface by oxidation of the monomers to form reactive radicals [32, 71, 72]. The characteristics of the polymeric film, i.e., thickness, porosity, and uniformity, can be controlled by the applied potential, current flow, and scanning speed as well as by additives within the precursor solution, temperature, and pH and ionic strength of the solution. Within a single step, this technique allows the growth of a conductive film from nanometers up to several hundreds of microns [56]. General characteristics of the electrode surface such as surface morphology, wettability, and electrical properties are equally influential [34, 68, 71, 72]. In contrast to most other methods, parameters change throughout the process, which is not limited to the concentration of the precursor molecules but especially also the conductivity in dependence of the increasing layer thickness. Overall, electrochemical deposition allows a highly controllable formation of the structure and properties of the conductive polymeric layer.

**Fig. 4:**
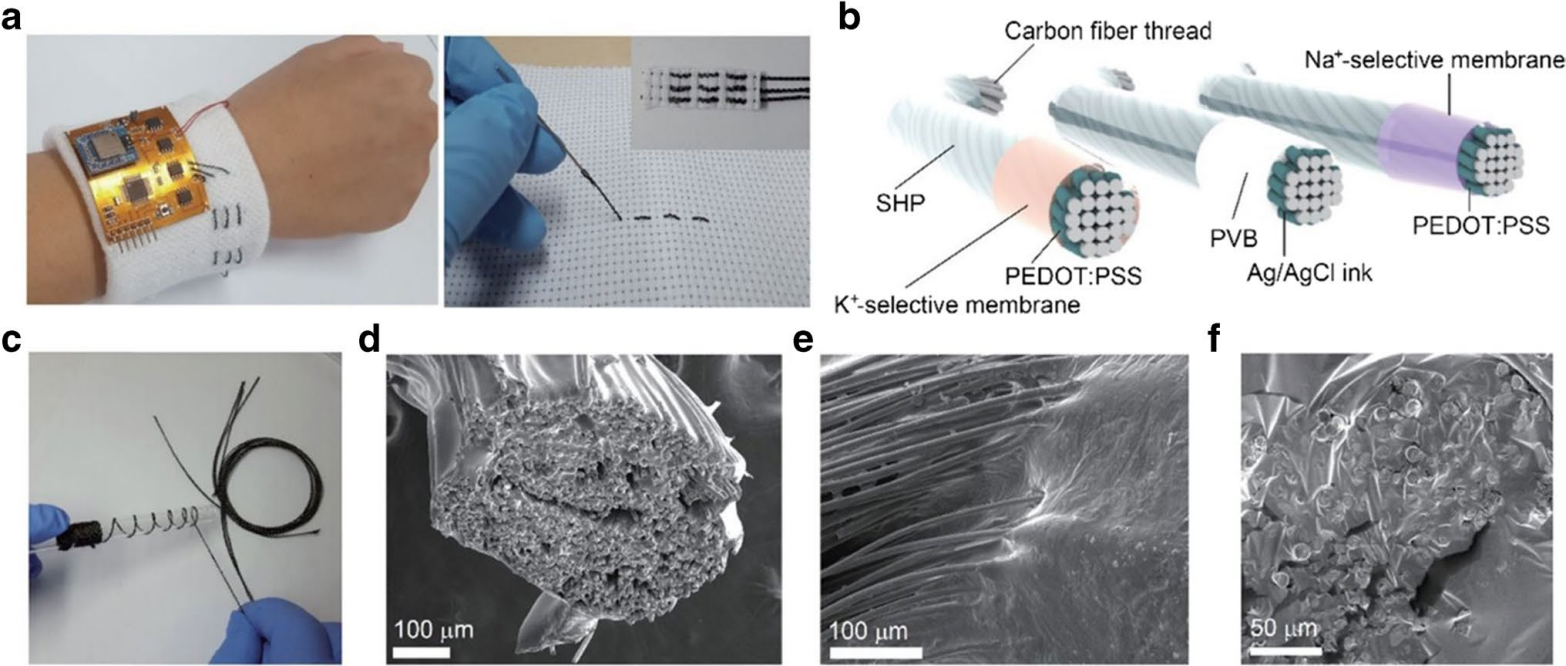
Yoon et al. present an electrodeposited PEDOT:PSS-based biocompatible polymer that is capable of self-healing via hydrogen bonding. Combination with carbon fiber thread (**c**) and ion-selective membranes (**b**) makes it a versatile new toolset for a wearable perspiration sensor which can be directly knitted into textiles (**a**). Via scanning electron microscopy images (**d** to **f**), the group proved the self-healing properties (reprinted with permission from [32], Copyright © 2021 American Chemical Society)

Many groups use electrodeposited conductive polymers to improve the electrochemical biosensor and chemosensor performance or add desirable features, where, e.g., thin films of polyaniline (PANI) could be optimized for optical and potentiometric pH sensors [33], and electrochemically depositing PEDOT membranes improved the LOD by two orders of magnitude for breast cancer biomarkers [12, 69]. Other researchers combined the electrochemical with other deposition methods, harnessing the strengths of the respective method for a polymer of choice. Here, Yoon et al. investigated autonomous self-healing sensors based on electrodeposited PEDOT:PSS carbon fiber threads for the preparation of wearable K^+^ and Na^+^ sweat sensors (Fig. 4) [32]. The conductive polymer itself is used as a solid contact transducer converting charge carriers from ions to electrons by the redox process of PEDOT:PSS in combination with a dip-coated ion-selective membrane. Cui et al. used the electrochemical technique for the deposition of a chitosan layer for the detection of organophosphate pesticides with immobilized acetylcholinesterase (AChE) [11]. Due to the positive charge of the non-conductive CS film, the negatively charged AChE/BSA mixture can furthermore be easily drop-coated subsequently to the film formation. The probe self-assembled homogeneously on the surface due to the electrostatic interactions and therefore overcame the disadvantage of inhomogeneity of the drop-coating technique.

### Strengths and weaknesses of the deposition techniques

The overall purpose and goal of any sensor modification through a film (gel or membrane) is to enable or improve its performance. Deposition techniques foremostly affect the film’s surface morphology and thickness and hence have to be wisely chosen. Furthermore, they determine the overall coating area and shapes achievable, which influences their potential for the production of large quantities of sensors such as in a commercialized product. Each method has its own strengths and weaknesses with respect to these parameters, which are summarized in Table 1. The other relevant properties of the polymer and/or hydrogel membrane(s) are mostly influenced by their chemical nature, such as mechanical stability, mesh size and cross-linking degree, concentration of the active ingredients (receptors and transducers), and adherence to their substrate.

Surface morphology plays a role regarding contact angle and therefore wettability of the sensor membrane, and may also influence resistance against fouling processes, albeit this latter property is most often addressed through additional protective membrane layers. The three methods of greatest interest here are solution casting, electrospinning, and electrodeposition as in all three cases the surface morphology can be influenced and even designed toward specific outcomes.

Membrane thickness influences sample molecule accumulation, diffusion, and hence reaction and response kinetics. Most techniques allow for easy adjustment of thicknesses in the nanometer to micrometer range, with the exception of knife coating where sub-micrometer heights are difficult to achieve. While drop coating and solution casting are also less suitable for thin membranes, spray coating, spin coating, and electrochemical deposition are the preferred methods to create sub-micrometer layers reproducibly. If a composition of multiple layers is planned, one should consider spray coating, spin coating, electrospinning, and electrochemical deposition for thinner membranes and knife coating for thicker membranes. While multilayering is possible also with dip coating, drop coating, and spin coating, these methods provide less reproducibility, and for solution casting, the multilayering fabrication process becomes laborious and time consuming. Furthermore, in the case of multilayered films, a combination of the deposition methods may be advantageous rather than sticking with one method, albeit the latter is easier on large-scale production. However, oftentimes, combinations of the more refined methods such as solution casting, electrospinning, or electrodeposition with the simpler methods may be a good choice, e.g., for generating an additional protective layer (i.e., an overcoat membrane) on top of a multilayered membrane.

Complicated shapes of a sensor are best addressed by solution casting because a mold can be perfectly adapted to the desired form the membrane should adopt. For spray coating and electrospinning of a dedicated shape, masks are required but more waste will be produced. Spin coating also has the potential to create certain geometries, e.g., by engraving the rotating disk. However, reproducible membranes will only arise as long as enough sensor cocktail is added and the membrane is cut into shape after annealing or drying. A rather new but very elegant method is to engrave the desired shape of the membrane by the use of a laser cutter or laser scriber. Those devices permit pre-defining a sensor shape exactly by a vector-based software. Laser scribing can be used to shape a membrane after any of the deposition methods described here, but the devices are expensive. Simpler, e.g., round, shapes can easily be obtained by using hole punchers or a toggle press from sensor layers created with any of the methods shown in Table 1.

In the case of affordability, lab vs. production scale, drop coating for small flat areas like electrodes and dip coating for fibers and sensor stripes remain optimal for inexpensive lab-scale fabrication. Both are very simple without special equipment needed, and useful for demonstrating a new sensor concept or proving its fundamental functionality. Drop coating on lab scale is the right choice when no special morphology is needed, and the film thickness does not play a major role. For increased production purposes and for the fabrication of large areas, knife coating, spray coating, and spin coating offer a straightforward approach when no special surface morphology is needed, as those methods will support role-to-role production [20, 54, 56]. Disadvantages like inhomogeneity of the membranes or batch-to-batch reproducibility especially on lab scale can be overcome by embedding reference dyes combined with ratiometric measurement strategies at two different wavelengths or with decay times, or by using other referencing methods [73].

If one has precious sensor cocktails to be deposited that, e.g., contain expensive biomolecules, a method that has a high deposition yield on the substrate and creates little waste will come into focus. Here, drop coating and solution casting are the best methods that can convert low volumes of sensor cocktail to the largest area of sensor membrane. Furthermore, for fragile biomolecules, pressure applied upon spray coating may negatively affect their structure, e.g., tertiary or quaternary structures of proteins or antibodies in the cocktail. Using electrospinning and electrochemical deposition poses the challenge that the biomolecule needs to be stable against the applied oxidation or reduction potentials.

## Conclusion and future perspective

Hydrogels and polymers are essential components of most sensors through which in vivo, continuous, and rapid analysis in an original (most complex) sample matrix is enabled. In fact, it has been known for decades that these are most often key to rendering a sensor functional not only in pristine buffer solutions but also in the murky real-world samples, where they are supposed to function after all. Much research is hence put toward the development of new, sophisticated and customized polymers and hydrogels [74]. Also, layered and blended polymer cocktails are investigated to provide an optimal sensing environment.

Yet, the importance of the deposition method to bring such layers to their full functionality is too often underestimated in academic research. In fact, most times, a critical sensor performance relies on the special features obtained through the deposition method chosen. Unfortunately, results obtained through other methods are seldom published, which makes quantitative comparison between methods difficult. Furthermore though, this dependence on a deposition or fabrication method may become the sole reason why lab-scale sensors will not be easily adapted to large-scale production, because their performance will inherently decrease.

Therefore, initial screening of various production methods is advisable already during the initial sensor development phase. Important points to be considered are requirements for surface morphologies, simple or complicated shaped (multi)layered membrane set-ups, and desired production scale, i.e., proof of a new principle or desired translation into mass production. It is clear that many dead-end research studies especially in bioanalytical sensors are preventable where the interplay between solvents needed for special polymers, for an effective deposition technique, and the delicate nature of the biorecognition element may not be well balanced. A change in deposition techniques may instead rapidly provide new avenues to be followed to obtain a new sensor membrane with the properties initially desired.
